# Comparative use of aqueous humour ^1^H NMR metabolomics and potassium concentration for PMI estimation in an animal model

**DOI:** 10.1007/s00414-020-02468-w

**Published:** 2020-11-20

**Authors:** Emanuela Locci, Matteo Stocchero, Rossella Gottardo, Fabio De-Giorgio, Roberto Demontis, Matteo Nioi, Alberto Chighine, Franco Tagliaro, Ernesto d’Aloja

**Affiliations:** 1 Department of Medical Sciences and Public Health, Section of Legal Medicine, University of Cagliari, Cittadella Universitaria di Monserrato, 09042 Monserrato, CA Italy; 2 Department of Women’s and Children’s Health, University of Padova, Padova, Italy; 3 Department of Diagnostics and Public Health, Unit of Forensic Medicine, University of Verona, Verona, Italy; 4 Department of Health Surveillance and Bioethics, Section of Legal Medicine, Catholic University of Rome, Rome, Italy; 5 Fondazione Policlinico Universitario A. Gemelli IRCCS , Rome, Italy; 6 Institute of Pharmacy and Translational Medicine, Sechenov First Medical University, Moscow, Russia

**Keywords:** Aqueous humour, Animal model, PMI, Potassium concentration, CIA, ^1^H NMR metabolomics

## Abstract

Estimation of the post-mortem interval (PMI) remains a matter of concern in the forensic scenario. Traditional and novel approaches are not yet able to fully address this issue, which relies on complex biological phenomena triggered by death. For this purpose, eye compartments may be chosen for experimental studies because they are more resistant to post-mortem modifications. Vitreous humour, in particular, has been extensively investigated, with potassium concentration ([K^+^]) being the marker that is better correlated with PMI estimation. Recently, a ^1^H nuclear magnetic resonance (NMR) metabolomic approach based on aqueous humour (AH) from an animal model was proposed for PMI estimation, resulting in a robust and validated regression model. Here we studied the variation in [K^+^] in the same experimental setup. [K^+^] was determined through capillary ion analysis (CIA) and a regression analysis was performed. Moreover, it was investigated whether the PMI information related to potassium could improve the metabolome predictive power in estimating the PMI. Interestingly, we found that a part of the metabolomic profile is able to explain most of the information carried by potassium, suggesting that the rise in both potassium and metabolite concentrations relies on a similar biological mechanism. In the first 24-h PMI window, the AH metabolomic profile shows greater predictive power than [K^+^] behaviour, suggesting its potential use as an additional tool for estimating the time since death.

**Supplementary Information:**

The online version contains supplementary material available at 10.1007/s00414-020-02468-w.

## Introduction

Accurate post-mortem interval (PMI) estimation is a challenging issue in forensic pathology. Traditional biochemical methods have been applied to identify metabolite changes after death in several biofluids [1–3]. Vitreous humour (VH) is the most studied fluid for post-mortem analysis given its correlation with ante-mortem serum composition and its longer stability compared with blood [4]. Potassium was the VH analyte most deeply investigated, and its time-dependent post-mortem increase is currently considered the most promising experimental predictor for PMI estimation [5–8].

After death potassium is mainly released from cells into VH because of increasing cellular hypoxia and consequent ATP depletion leading to a loss of selective membrane ion permeability. Studies have mainly focused on applying a regression analysis to find the best linear relationship between the variation in [K^+^] and the PMI. Confidence intervals were calculated up to 120 h after death. Several analytical and statistical approaches have been reported, but no univocal regression line for PMI estimation has been obtained. The lack of reproducibility is related to the cause of death, the duration of the agonal period, the external temperature, the cadaver putrefaction, pathologic conditions, the VH pre-treatment before analysis and the analytical platform. All these factors may affect both the intercept and the slope of the regression line, leading to weakly reproducible interlaboratory data precluding the translation of a general validated method for routine application in real cases [9].

Attempts to combine the results obtained from various laboratories were reported [10]. Inverse prediction procedures, and nonlinear regression analysis (Loess smooth curve) were considered, and prediction errors were calculated by cross-validation. A combination of the results of six independent studies yielded a more precise PMI estimation, with a 95% confidence limit of 1 h in the early and 10 h in the late post-mortem period, over a time window of 110 h. However, a re-evaluation of this approach using a new random VH sample of 492 cases revealed that only 153 cases showed a predicted PMI within the confidence interval for the given [K^+^] and that the remaining 339 cases laid outside this interval with a systematic PMI overestimation [11]. Therefore, the Lange combined approach accuracy could not be confirmed.

Capillary ion analysis (CIA) [12] has been recently proposed to determine [K^+^]. CIA showed several advantages, including the need for a limited sample volume and the chance to overcome interferences with other cations, but, due to high extrapolation errors in early PMIs, it is suitable for estimating PMIs higher than 24 h.

A combination of vitreous potassium with other biochemical components, such hypoxanthine, or external parameters, such as ambient temperature, was proposed by different authors to improve the precision of PMI determination [13, 14].

Very recently, ^1^H nuclear magnetic resonance (NMR) metabolomics has been proposed as a tool for PMI estimation based on the analysis of the global metabolic profile of a biofluid [15–18]. Metabolomics represents the quali-quantitative study of the low-molecular-weight metabolites in a biofluid or a tissue and their modifications under any pathophysiological stimuli, including death. ^1^H NMR, despite its low intrinsic sensitivity (low micromolar range), is a rapid and robust tool that allows us to obtain the global profile of a biofluid, in a non-destructive way, without extensive sample extraction and derivatization.

Ocular fluids have been recognized as the most suitable candidates for studying post-mortem modifications even from a metabolomic point of view. The majority of the studies describe modifications occurring after death without proposing appropriate prediction models. We recently employed ovine aqueous humour (AH) ^1^H NMR metabolomics to generate and validate a regression model for PMI estimation [19]. A total of 59 samples were collected at different times after death up to 24 h (from 118 to 1429 min) from very homogeneous animals. Single ocular sampling was performed to avoid bacterial contamination. Half of the eyes were kept with the eyelids sealed and the other half with the eyelids open. A multivariate calibration model was built based on the AH metabolomic profile of 38 samples and validated in prediction by the use of an independent test set of 21 additional samples. The prediction error over the entire temporal window was estimated to be approximately 100 min. While the model indicated taurine, choline and succinate as the best PMI biomarker candidates, the global profile was more accurate for PMI prediction than the models generated from any single metabolite. Indeed, the power of metabolomics relies on the simultaneous profiling of multiple and interacting metabolites that are able to intercept a complex and multivariate process such as death than the use of a single or a few markers. The effect of the eye condition (open or closed) on the AH metabolome was negligible with respect to the strong modifications occurring during the post-mortem period.

It has been shown that the metabolite levels in AH can give at least as good estimation of PMI as those in VH with the additional advantage of a residual protein, lipid and cellular content that allows us to perform ^1^H NMR without sample extraction [16]. The only drawback is that AH has a smaller volume and a higher evaporation rate than VH, which may hamper the analysis at late PMIs. Only few studies compared AH and VH post-mortem chemistry, most of them were conducted on animal models [20 and refs therein]. In the work of Butler et al., several analytes were simultaneously analysed in aqueous and vitreous fluids collected from 83 autopsies (PMI window = 5–69 h). A consistent correlation in the first 48 h post-mortem between AH and VH was observed, and the authors concluded that AH may be a viable substitute for VH for post-mortem chemistry. Potassium showed the best correlation between the two biofluids, regardless of early or late PMIs and adult or paediatric population. Very recently a metabolomic approach on human AH, VH and serum confirmed that AH is a viable biofluid for the estimation of PMI [21].

The objective of the present study was to perform a PMI regression analysis based on aqueous [K^+^] variation with the aim of comparing the results with those previously obtained by ^1^H NMR metabolomics, provided that vitreous potassium is currently considered to be the best experimental predictor for PMI estimation. To this aim, [K^+^] was measured by CIA on the same ovine AH samples used in the metabolomic approach. As previously done, a regression model was built and validated by the use of an independent set of AH samples. The accuracy of the models obtained by two different analytical methods was compared. In addition, the combined use of both approaches was tested with the perspective of improving PMI estimation precision and accuracy.

## Materials and methods

### Sample collection and preparation

The experimental protocol was previously described [19]. AH samples were collected from 36 sheep (*Ovis aries*) heads of young adult females belonging to the same herd obtained from a local slaughterhouse after animal sacrifice. Sheep heads are commonly discarded so ethical approval was not necessary. Heads were transported to the institute morgue and stored at a controlled humidity and temperature (50 ± 5% and 25 ± 2 °C, respectively). Approximately 2 h were needed to transport the heads to the morgue, where AH sampling was immediately started. To introduce a possible confounding factor, samples were collected from open and closed eyes. In particular, for each head, one eye was maintained open by surgical removal of the eyelids, and the opposite was maintained closed through surgical suture of the eyelids (until the moment of AH collection). AH was withdrawn from the anterior chamber through a corneal puncture on the sclerocorneal limbus, using a 5-ml syringe G22. Samples were collected every hour, starting from 2 until 24 h after death (in the exact range 118 – 1429 min). A single sampling was performed in each eye to avoid bacterial contamination, which may influence the AH metabolic composition. After collection, samples were centrifuged (13,000 g for 5 min) and immediately frozen at −80 °C.

By using a stratified random selection procedure based on the different ranges of PMI, 24 heads were selected for composing the training set, while the remaining 12 heads were used for the validation test set. Thirteen specimens were discarded, because of contamination or paucity of the sample. The final dataset comprised 38 training set and 21 test set samples. In both data sets the same temporal pace and window were maintained, and a constant open/closed eye sampling ratio was assured. As a result, the factors PMI and eye condition were orthogonal in the training set generating an orthogonal design matrix for a linear model with interaction.

### CIA experiments for potassium determination

#### Standards and chemicals

Standard solutions of potassium (K^+^) and barium (Ba^2+^) were prepared from AnalaR salts (KCl and BaCl_2_) (Merck, Darmstadt, Germany). 18-crown-6 ether (99% pure) and α-hydroxybutyric acid (HIBA) (99% pure) were obtained from Aldrich (Milan, Italy); imidazole (99% pure) and glacial acetic acid were obtained from Sigma (St. Louis, MO, USA). All chemicals were of analytical-reagent grade. Ultrapure water was obtained by an ELGA VEOLIA (Lane End, High Wycombe, UK) water purification system.

#### Instrumentation

All experiments were performed using a P/ACE MDQ Capillary Electrophoresis System (Beckman, Fullerton, CA, USA) equipped with a UV filter detector set at 214 nm wavelength, with indirect detection. In all the experiments, untreated fused-silica capillaries (75 μm I.D., 50 cm effective length; Beckman) were used with a detection window of 200 x 100 μm. The capillary was thermostated at 25 °C. Beckman P/ACE Station (version 8.0) software was used for instrument control, data acquisition and processing. Separations were performed as described in Palacio et al. [22]. Briefly, the running buffer was composed of 5-mM imidazole, 6-mM HIBA and 5-mM 18-crown-6 ether adjusted to pH 4.5 with acetic acid. Constant voltage runs were performed in all experiments by applying a field of 500 V/cm. The analytes were injected at the anodic end of the capillary at 0.5 psi for 10 s. Prior to analysis, all samples were diluted 1:20 with a 40 μg/mL solution of BaCl_2_ (internal standard). Between consecutive runs, the capillary was washed with water (3 min) and then with the running buffer (2 min).

### Statistical data analysis

Regression analysis was based on multiple linear regression (MLR) and projection to latent structure regression. Specifically, orthogonally constrained projection to latent structures (oCPLS2) [23] was applied to model PMI to remove the effects of the factor open/closed eye on the regression model, whereas post-transformed PLS2 (ptPLS2) [24, 25] was used to model [K^+^] on the basis of AH metabolite concentrations. The selectivity ratio (SR) was used to interpret the multivariate regression model [26]. Fivefold cross-validation was performed to optimize the model parameters. A permutation test on the response (1000 random permutations) was applied to highlight the presence of overfitting in the case of multivariate models according to good practice for model building. Suitable in-house functions implemented by the R 3.3.2 platform (R Foundation for Statistical Computing) were built for post-transformation of PLS2, oCPLS2 and MLR-based analysis.

## Results

### CIA [K^+^] determination

[K^+^] levels were determined in 59 AH samples collected at different PMI, ranging from 118 to 1429 min. The potassium concentrations ranged from 9.46 to 40.81 mM, the accuracy of the method being 7%.

### [K^+^] vs PMI regression analysis

To maintain consistency with our previous metabolomic study, the same distribution of the 59 AH samples in the training and test sets was maintained. In particular, 38 samples with PMI ranging from 118 to 1350 min were used for the training set. Half of them were collected from open eyes, and the other half were collected from closed eyes. The remaining 21 samples with PMI from 138 to 1429 min constituted the test set (10 from open and 11 from closed eyes).

First, in the training set, we investigated the relationships between [K^+^] and the two factors PMI and eye condition (EYE, open and closed). An MLR model was built using the following equation:

1 $$ {\left[{\mathrm{K}}^{+}\right]}^{-0.5}=\mathrm{a}\ \mathrm{PMI}+\mathrm{b}\ \mathrm{EYE}+\mathrm{c}\ \left(\mathrm{PMI}\ast \mathrm{EYE}\right)+\mathrm{d}+\upvarepsilon $$

where the term PMI*EYE has been introduced to take into account the interaction between PMI and EYE and ε is the residual term. The exponent −0.5 of [K^+^] has been estimated by the Box-Cox method to guarantee normally distributed residuals. The model showed a goodness-of-fit (R^2^Y) equal to 0.76 and Q^2^Y = 0.71 (i.e. R^2^Y calculated by fivefold cross-validation). The analysis of variance led to a *p* value less than 0.001. The parameters of the model are reported in Table 1. The model indicated a significant variation of [K^+^] as a function of PMI (*p* value < 0.05), whereas the effects of the eye condition and the interaction factor PMI*EYE were nonsignificant (*p* value > 0.05). This indicates a much stronger effect of PMI on the variation in [K^+^] than the effect of the eye condition, which can in principle be neglected. Provided that, we applied a nonlinear fitting procedure based on the following model:

**Table 1:** Parameters of the MLR model of [K^+^] as a function of PMI, EYE and PMI*EYE (Eq. 1)

Parameter		Value	Standard error	*p* value
a		−0.06	0.01	< 0.001
b	EYE(open)	−0.0004	0.004	0.92
	EYE(closed)	0.0004	0.004	0.92
c	PMI*EYE(open)	−0.0001	0.0002	0.60
	PMI*EYE(closed)	0.0001	0.0002	0.60
d		0.245	0.004	< 0.001

2 $$ \mathrm{PMI}=\mathrm{a}\ {\left[{\mathrm{K}}^{+}\right]}^{\upalpha}+\mathrm{d}+\upvarepsilon $$

where the residual term ε is normally distributed. The parameters have been estimated by least-squares maximizing Q^2^Y and assuming values of α between −3 and 3. The behaviour of Q^2^Y is reported in Fig. S1 in the Supplementary Material. The best model was obtained for α = −0.50 and gave R^2^Y = 0.76 and Q^2^Y = 0.75 (corresponding to a standard deviation error in cross-validation SDECV = 189 min) (see Fig. 1). The analysis of variance provided a *p* value < 0.001. The parameters of the model are reported in Table 2. The standard deviation errors in calculation (SDEC) and prediction (SDEP) are reported in Table 3. They resulted in 209 min for PMI lower than 500 min, 164 min for PMI from 500 to 1000 min and 190 min for PMI longer than 1000 min (global SDEC = 186 min). The prediction with the test set provided the following estimations of the standard deviation error in prediction (SDEP): 296 min for PMI lower than 500 min, 184 min for PMI from 500 to 1000 min and 134 min for PMI longer than 1000 min (global SDEP = 210 min). For the sake of comparison, the corresponding values obtained for the model previously published using the AH metabolomic profiles [19] are also reported in Table 3. Thus, the models generated with potassium are less efficient both in calculation and prediction than the model based on the AH metabolite concentrations.

**Fig. 1:**
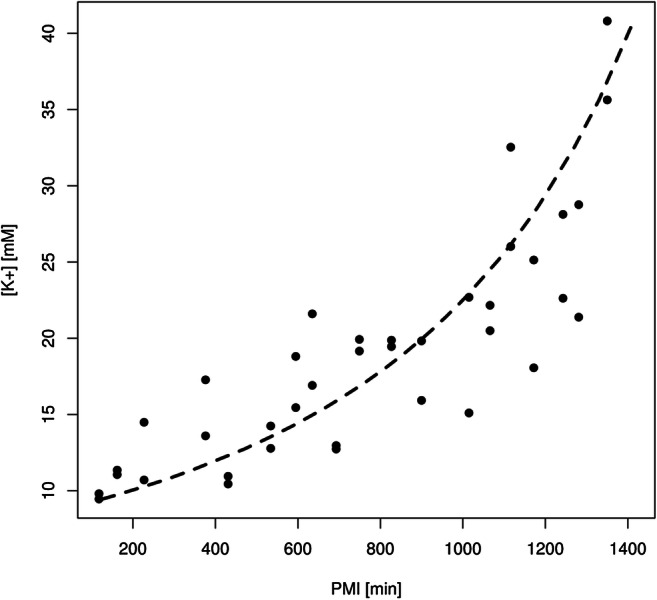
Regression model of PMI vs [K^+^]: the dashed line represents the curve that best fits the data

**Table 2:** Parameters of the best regression model (α = −0.50) based on Eq. (2)

Parameter	Value	Standard error	*p* value
d	2600	170	< 0.001
a	−7600	720	< 0.001

**Table 3:** Standard deviation errors of the model obtained by using Eq. (2) and the previously reported ^1^H NMR metabolomics data

Model	SD error	PMI range (min)			
		118 < PMI < 1429	PMI < 500	500 < PMI < 1000	PMI > 1000
Eq. (2)	SDEC	186	209	164	190
	SDEP	210	296	184	134
^1^H NMR [19]	SDEC	88	52	97	99
	SDEP	99	59	104	118

### [K^+^] and AH metabolite concentrations vs PMI

**Fig. 2:**
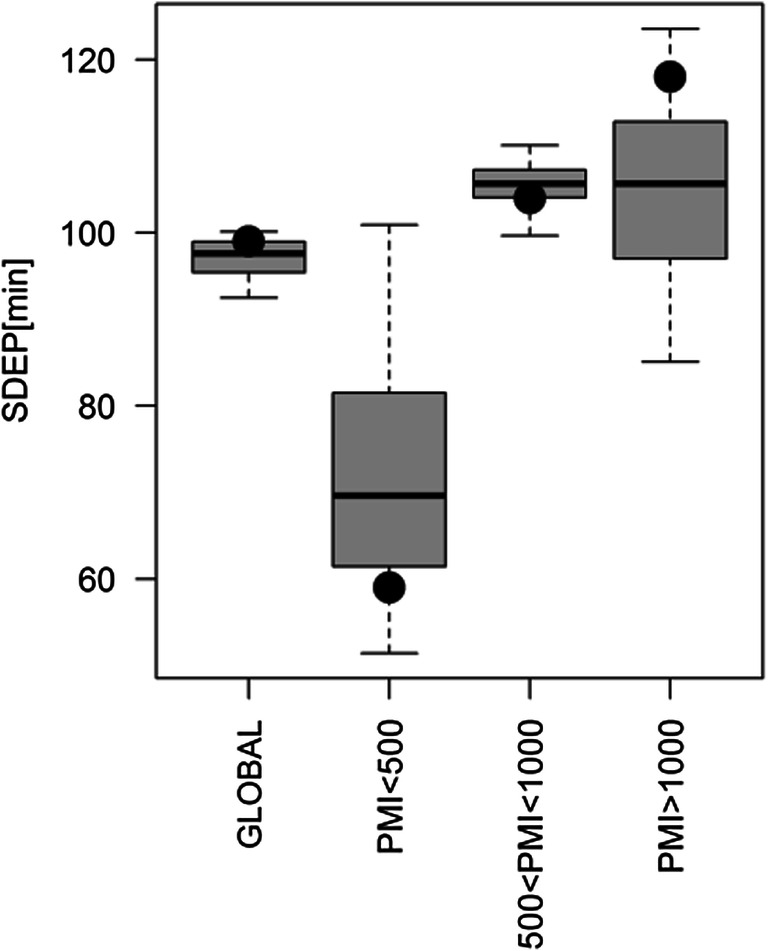
Multivariate regression models based on oCPLS2 where both [K^+^] and AH metabolite concentrations are used to estimate PMI. The box plots describe the distribution of SDEP, for the models with SDEP less than the 5th percentile, for the levels of PMI of interest (the black circles represent the values obtained for the model described in Locci et al. [19])

Since Locci et al. [19] proved that AH metabolite concentrations satisfactorily model the behaviour of PMI, we evaluated whether the combination of [K^+^] and AH metabolomic profiles can improve the power of the PMI prediction. To achieve this aim, the following regression equation was considered:

3 $$ {\mathrm{PMI}}^{\upbeta}=\left(\mathrm{X}{\left[{\mathrm{K}}^{+}\right]}^{\upalpha}\right)\ {\mathrm{b}}_{\mathrm{oCPLS}2}+\mathrm{e} $$

where β and α are model parameters, (X[K^+^]^α^) is the block obtained juxtaposing the block X of the metabolite concentrations and the [K^+^] raised to the power α and e is the vector of the residuals. The vector of the regression coefficients b_oCPLS2_ has been calculated by oCPLS2 with the eye condition as a constraint. Exploring the space of all the possible combinations of β and α and applying different types of scaling factors, we generated a set of models with different performances in prediction. Interestingly, the multivariate calibration model previously published that used only the AH metabolomic profiles showed similar performance to the best models obtained in this study, proving that the addition of [K^+^] (or its power) as predictor does not improve the power in prediction. This can be clearly seen in Fig. 2, where the distributions of SDEP obtained considering the models with SDEP less than the 5th percentile and the three levels of interest for PMI are reported as box plots. The model described in Locci et al. [19] (black circle in Fig. 2) that used only the AH metabolite concentrations showed performance similar to that of the best models obtained including [K^+^] (or its power) as a predictor. More technical details about the data analysis are reported in the Supplementary Material.

### [K^+^] vs AH metabolite concentrations

To explain why the addition of [K^+^] is not relevant for improving the performance of the model based on AH metabolomic profiles, the relationships between potassium and the metabolite concentrations were investigated considering the model:

4 $$ {\left[{\mathrm{K}}^{+}\right]}^{\upalpha}=\mathrm{X}\ {\mathrm{b}}_{\mathrm{ptPLS}2}+\mathrm{e} $$

where α is a model parameter in the range [−3,3], X is the block of the metabolite concentrations, e is the vector of the residuals and ptPLS2 was applied to calculate the vector of the regression coefficients b_ptPLS2_. Independent of the value of the power α, the metabolite concentration was able to satisfactorily explain the behaviour of [K^+^]. Only a small part of the information contained in [K^+^] was not modelled by the AH metabolic profile. However, this information was unsuitable to interpret the behaviour of PMI. Thus, we can conclude that the variation in [K^+^]^α^ that is useful to explain PMI can be modelled by a linear combination of the metabolite concentrations and that [K^+^]^α^ should not add information when it is coupled with the data set composed of the AH metabolites (as previously observed). Moreover, the analysis of the spectrum of SR allowed us to identify two significant metabolites (α = 0.05), choline and taurine that are always the most relevant to explain [K^+^] for all ptPLS2 models and succinate which appears to be significative in nearly the 70% of the models. More technical details about the data analysis can be found in the Supplementary Material.

## Discussion

A major aim of this work was to analyse the post-mortem related increase in AH potassium concentration. This fluid is seldom analysed in a forensic context, as it begins to change in volume early after death. For this reason, its value as a bio-specimen for forensic investigation of time since death seems to have limited predictive value. Although widely employed in the analysis of post-mortem modification of the VH, potassium behaviour in the AH, to the best of our knowledge, has never been investigated as a tool to estimate PMI. Our secondary interest was also to compare the potassium approach towards our recently proposed metabolomic profiling approach as a PMI predictive tool on the same specimen. The experimental animal setup was a priori suitable to create a model; to control the biological effects of one variable among the others, namely, the eye condition, which may affect biological transformation related to death; and to test such a model against an independent prediction set to calculate the standard deviation errors in calculation and prediction.

The best regression model of our experimental AH potassium concentrations, measured at different PMIs, indicated a nonlinear potassium variation as a function of PMI over the sampled 24-htime window. The effect of the eye condition (eye maintained open or closed) was negligible, and Eq. (2) resulted in the model with the best regression parameters, which are reported in Table 2. For potassium behaviour in vitreous humour, the majority of the relevant literature proposed a linear model but even a nonlinear one was suggested as a function of PMI, with a faster rise in the first few hours than after 24 h after death [4]. The predictive ability of the AH potassium model calculated by the use of the test set samples indicated that potassium is less performant than the ^1^H NMR metabolomic profile for PMI estimation, since the errors in prediction over the 24-h period were 210 versus 99 min, respectively. Interestingly, while the prediction errors determined by ^1^H NMR metabolomics in the three predefined PMI ranges increased with increasing PMI, an opposite trend was observed in the potassium model. Increasing prediction errors were interpreted as related to the concomitant existence of overlapping microbial metabolism at higher PMIs, a hypothesis that has yet to be proven but is suggested by the late appearance of metabolites mainly related to microbial metabolism (viz. acetate and dimethylsulfone). Decreasing potassium errors are in line with what is observed with vitreous potassium, where PMI extrapolation errors were observed to decrease with increasing PMI, indicating increased suitability for forensic casework analysis later than the 24-hperiod after death [4, 12].

Considering that both ^1^H NMR metabolomics and potassium explain a large part of the total variance of PMI (more than 75% in either case, resulting from both model statistical parameters R^2^Y), from the perspective of improving the power in the PMI prediction, we tried to combine the two independent results, i.e. AH [K^+^] and metabolomic profiles. To this aim, we considered the variation in potassium concentration as an additional variable (parameter) to be used for building the PMI regression model (see Eq. 3). However, this combined approach did not provide any advantage in forecasting, showing a lower predictive ability than the model built solely with ^1^H NMR metabolomics. In particular, a large number of models (450) were generated optimizing the number of latent variables and the scaling factors to maximize the predictive ability (Q^2^Y) of the resulting model (see Fig. S2 in Supplementary Material). The box plots of the distributions of the errors in prediction of the best regression models (SDEP less than the 5th percentile) indicate that errors, both in the entire and in the three PMI ranges of interest, were comparable to, or higher than those determined using metabolomic data (Fig. 2). These results suggest that the contribution of potassium concentration to metabolomics does not improve the accuracy of the PMI estimation but worsens it in some ways. We found a potential explanation for this behaviour by investigating the relationship between potassium and the metabolite concentrations determined by NMR (see Eq. 4). Specifically, a number of different models were built by varying several features, such as the number of latent variables and the scaling factors used to maximize the statistical parameter Q^2^Y, i.e. the estimation of the predictive power. For all the obtained models, the results showed that all the information attainable from potassium concentration and useful for PMI determination were already included in the NMR metabolomic profile. It is worth noting that, for all the obtained models, approximately 20% of the total variance in the profile explained more than 60% of the total variance of potassium, the residual 40% being orthogonal to PMI, and so non-informative for its determination. Choline and taurine were the metabolites most significant in explaining the potassium variation in the majority of the obtained models. On the other hand, the vast majority (up to 80%) of the total variance of metabolites that is not linked to potassium is still informative for PMI, and this is why the NMR model alone showed the highest predictive ability. In summary, under our experimental conditions, all the information contained in potassium variation is already included in the metabolomic profile and AH potassium does not add any additional information to better predict PMI. Although the goal of this study was not to speculate on the complex biological modifications occurring post-mortem, a possible explanation of this interesting result could be given if the post-mortem rise in potassium concentration is related to the variations in the NMR determined metabolite concentrations, with particular attention being paid to those metabolites that are contributing the most to explain both PMI and potassium increase, i.e. taurine and choline. Notably, these two metabolites together with succinate (represented in nearly 70% of the models of potassium) are the most relevant metabolites in explaining PMI in the metabolomic model. Although the design of the experiment did not include this endpoint and the data were underpowered to address this issue, a shared metabolic trajectory may be hypothesized. Both modifications rely on the progressive fading of vital energetic phenomena, being the residual ability of the corpse and of its organs to cope with the death processes related to a time-dependent energetic breakdown. It may be supposed that in a metabolomic profile modification, a relevant contribution may be due to progressive overlap of the microbial metabolism (a co-metabolism), while the potassium modification could be due to a pure fully human-driven phenomenon, associated with the ongoing failure of the energy-dependent activities. If so, metabolomic profiling may be more informative and accurate in an early or a late period after death, whenever an absence, or a surge, of the microbiome plays a relevant role. After the cessation of residual cellular ATP-dependent activities, which may maintain an active concentration difference for some time, the potassium concentration in AH and VH should rely on the existence of an osmotic gradient between interfacing compartments, with a progressive trend towards the steady state. Although this phenomenon may be indirectly influenced by the superposition of microbial metabolism, this hypothesis seems to be, from a mechanistic point of view, less considerable than the effects being caused by the whole metabolome. As the underlying mechanism is partially shared by the two trajectories, it appears plausible that the majority of the information provided by the potassium changes is contained in a minimal part of the metabolomic profile variation.

This study presents some limitations. The explored PMI time window was limited to the first 24 h, but this is intrinsically related to the biofluid under study, since it is very difficult to collect AH samples at a PMI longer than 24 h due to post-mortem dehydration progression over time. This may hinder the application of this approach in routine forensic caseworks where higher PMIs are generally under scrutiny. Moreover, the advantage of using an animal model with highly controlled experimental conditions also represents a limitation, since all the animals were similar and in good health, the cause of death was homogeneous and all the real-world variability was not taken into account. We are also aware that NMR facilities are not common in forensic laboratories, but metabolic profiling could also be performed with GC- or LC-MS equipment provided the development of appropriate experimental protocols. Finally, the translation of our results to humans is not immediate.

Despite the abovementioned limitations, we obtained robust regression models for PMI estimation (potassium and metabolomics), both of which were validated by the use of an independent prediction set. A comparison using the same animal data set showed that a multiparametric metabolomic profiling approach had higher predictive power than approaches with one or a few parameters. The proposed approach represents one more analytical tool available to address the issue of time since death estimation.

## Supplementary Information

ESM 1 (PDF 294 kb)

## Data Availability

The datasets generated during and/or analysed during the current study are available from the corresponding author on reasonable request.
