# Supplementary material for: Comparative use of aqueous humour 1H NMR metabolomics and potassium concentration for PMI estimation in an animal model
Source: Int J Legal Med. 2020 Nov 20;135(3):845–52. doi: 10.1007/s00414-020-02468-w (PMC8036180; doi:10.1007/s00414-020-02468-w)
Supplement: Supplementary file 1 — (PDF 294 kb) [file 414_2020_2468_MOESM1_ESM.pdf]

Supplementary Material

Comparative use of aqueous humour ^{1}H NMR metabolomics and potassium concentration for PMI estimation in an animal model

Emanuela Locci[1,*], Matteo Stocchero[2], Rossella Gottardo[3], Fabio De-Giorgio[4], Roberto Demontis[1], Matteo Nioi[1], Alberto Chighine[1], Franco Tagliaro[3,5], Ernesto d'Aloja[1]

[1]Department of Medical Sciences and Public Health, Section of Legal Medicine, University of Cagliari, Cagliari, Italy
[2]Department of Women's and Children's Health, University of Padova, Padova, Italy
[3]Department of Diagnostics and Public Health, Unit of Forensic Medicine, University of Verona, Verona, Italy
[4]Institute of Public Health, Section of Legal Medicine, Catholic University of Rome, Rome, Italy
[5]Institute of Pharmacy and Translational Medicine, Sechenov First Medical University, Moscow, Russia

*Corresponding author e-mail: elocci@unica.it

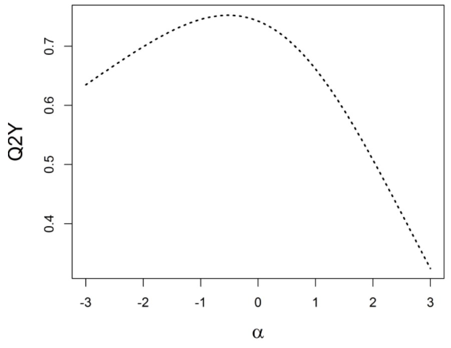

Fig. S1 Regression model of PMI vs [K^+]: plot of Q^2Y vs α used to estimate the optimal value of the power α

[K^+] and AH metabolite concentrations vs PMI

To evaluate whether the combination of [K^+] and AH metabolite concentration can improve the performance of the model of PMI obtained considering only the AH metabolite concentration, oCPLS2 was applied to model PMI according to eq. 3.

oCPLS2 is a regression technique that allows the inclusion of constraints in the framework of PLS. Specifically, the matrix of the constraints was set codifying the qualitative factor open/closed eye as two dummy variables with 0 and 1 depending on the state of the eye. Three scaling factors were considered: Pareto scaling, unit variance scaling and no scaling. Data were mean-centred. The number of latent variables and the scaling factor se were determined maximizing Q^2Y. In Fig. S2 the global SDEP obtained predicting the test set is reported for the models obtained assuming a β between 0 and 3, and an α between -3 and 3.

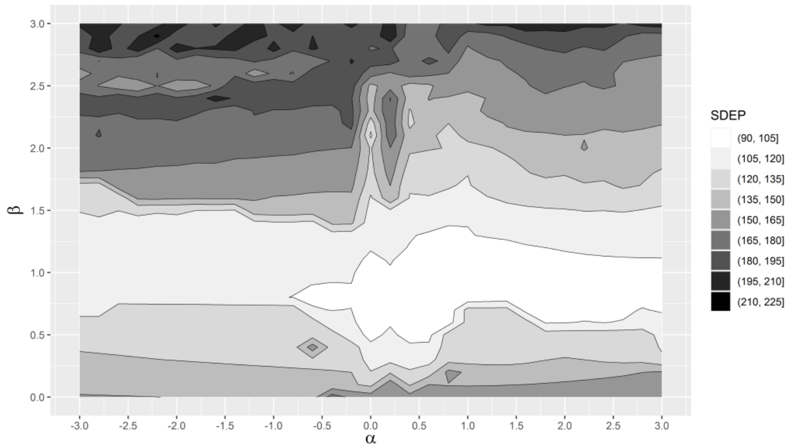

Fig. S2 Multivariate regression models based on oCPLS2 where both [K^+] and AH metabolite concentrations are used to estimate PMI. The contour plot shows the behaviour of the global SDEP with respect the parameters β and α

$[K^+]$ vs AH metabolite concentrations

To investigate the relationships between $[K^+]$ and AH metabolite concentration ptPLS2 was applied with the objective of generating a multivariate calibration model.

Three scaling factors were considered: Pareto scaling, unit variance scaling and no scaling. Data were mean-centred. The number of latent variables and the scaling were determined maximizing Q^2Y. As reported in Fig. S3a, the total explained variance of $[K^+]^\alpha$ was greater than 0.60 for all the models with α in [-3, 3] and the fraction of the total variance of the X-block of the metabolites used to model the response was approximately equal to 22% independently of α. Moreover, the unexplained variance of $[K^+]^\alpha$, which is orthogonal to the block of the metabolites, showed a poor correlation with PMI for all the values of α (Fig. S3b).

These results proved that the variation in $[K^+]^\alpha$ useful to explain PMI can be modelled by a linear combination of the metabolite concentrations and that $[K^+]^\alpha$ should not add information when it is coupled with the data set composed of the AH metabolites (as previously observed).

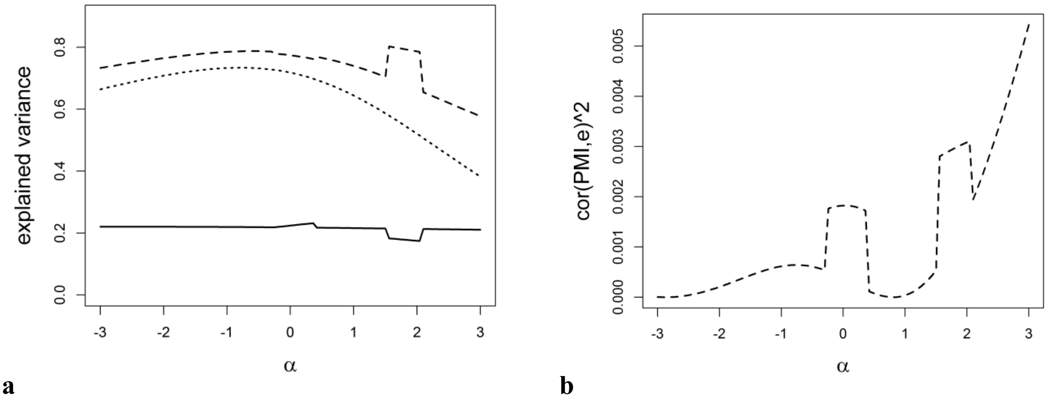

a **b**

Fig. S3 Investigation of the relationships between $[K^+]$ and AH metabolite concentrations. Panel A: ptPLS2 models considering α between -3 and 3. The dashed line indicates R^2Y, the dotted line the best Q^2Y and the black line the fraction of the total variance of the X-block used to explain $[K^+]$. Panel B: square of the Pearson correlation coefficient of PMI and the residuals of $[K^+]$ (i.e., the term e in eq. 4) vs α
